# Digital Skills to Improve Levels of Care and Renew Health Care Professions

**DOI:** 10.2196/58743

**Published:** 2024-05-01

**Authors:** Massimo De Martinis, Lia Ginaldi

**Affiliations:** 1 Department of Life, Health and Environmental Sciences, University of L’Aquila, L'Aquila, Italy; 2 Long-Term Care Unit, Maria SS dello Splendore, AUSL 04 Teramo, Giulianova, Italy; 3 UniCamillus-Saint Camillus International University of Health Sciences, Rome, Italy; 4 School of Nursing, Teramo Hub, University of L’Aquila, L'Aquila, Italy; 5 Allergy and Clinical Immunology Unit, Center for the Diagnosis and Treatment of Osteoporosis, AUSL 04 Teramo, Teramo, Italy

**Keywords:** digital competence, telehealth, nursing, health care workforce, health care professionals, informatics, education, curriculum, interdisciplinary education, health care education

We read with great interest the article by Rettinger et al [1], “Telehealth education in allied health care and nursing: web-based cross-sectional survey of students’ perceived knowledge, skills, attitudes, and experience,” recently published in *JMIR Medical Education*.

The authors, addressing an extremely current topic, highlight the need to integrate telehealth into health care education curricula. More generally, we think that the development of digital competence is essential for all health care professionals. The digitalization of care processes requires ever-greater digital skills to ensure high-level care suited to current knowledge. Another recent investigation [2] summarizes the educational intervention methods that have been implemented to develop digital competence and the effects of these educational interventions on health care workforce; this study suggests the best method for enhancing the digital skills of nurses and allied professionals in the context of continuing professional education. This research turned attention to the active workforce, who need to adapt their knowledge to renewed working contexts where digital technology is forcefully entering. However, we must note, as emphasized by Rettinger et al [1], that our curricula often neglect the need to equip health care degree students with adequate digital skills. We observe that few of our students are keeping up with the development of technology. Digital skills can range from the simplest to the most sophisticated technological applications commonly used in a hospital environment, including the use of virtual simulators and extending to artificial intelligence, which, especially in the coming years, will become a precious tool for improving care processes [3]. Even for delivering high-quality care in digitally enabled health care environments, nursing informatics competency is a required core competency [4]. In light of this, it would be necessary to introduce programs dedicated to the acquisition of these skills into our study courses; these programs could be spread across all curricular disciplines. To achieve these objectives, it is necessary to ensure that teachers have the necessary skills in this field or have the ability to acquire them to pass them on to their students. We are well aware that the nursing profession is going through a period of crisis and that it is essential to implement all available forces and strategies to renew it, making it attractive and satisfying again [5]. There are numerous proposals for this renewal, and they must also address the active workforce; however, the updating of the study contents for degree courses in health professions must be one of the first and fundamental steps to achieve these results. The acquisition of adequate digital skills is a necessity that can no longer be postponed to train professionals capable of providing the best levels of care possible today.
